# Associations between Fatty Acid Intakes and Plasma Phospholipid Fatty Acid Concentrations in the European Prospective Investigation into Cancer and Nutrition

**DOI:** 10.3390/nu15173695

**Published:** 2023-08-23

**Authors:** Inge Huybrechts, Inarie Jacobs, Elom K. Aglago, Sahar Yammine, Michèle Matta, Julie A. Schmidt, Corinne Casagrande, Geneviève Nicolas, Carine Biessy, Heleen Van Puyvelde, Augustin Scalbert, Jeroen W. G. Derksen, Yvonne T. van der Schouw, Sara Grioni, Pilar Amiano, Jytte Halkjær, Anne Tjønneland, José M. Huerta, Leila Luján-Barroso, Domenico Palli, Marc J. Gunter, Aurora Perez-Cornago, Véronique Chajès

**Affiliations:** 1 Nutrition and Metabolism Branch, International Agency for Research on Cancer, CEDEX 07, 69366 Lyon, France; jacobsi@iarc.who.int (I.J.); aglagoelom@gmail.com (E.K.A.); saharyammine@hotmail.com (S.Y.); mattam@iarc.who.int (M.M.); casagrande.masuyer@gmail.com (C.C.); nicolasg@iarc.who.int (G.N.); biessyc@iarc.who.int (C.B.); heleenvanpuyvelde@gmail.com (H.V.P.); scalbertaugustin@gmail.com (A.S.); gunterm@iarc.who.int (M.J.G.); chajesv@iarc.who.int (V.C.); 2 Cancer Epidemiology Unit, Nuffield Department of Population Health, University of Oxford, Oxford OX3 7LF, UK; js@clin.au.dk (J.A.S.); aurora.perez-cornago@ndph.ox.ac.uk (A.P.-C.); 3 Department of Clinical Epidemiology, Department of Clinical Medicine, Aarhus University Hospital, Aarhus University, 8200 Aarhus, Denmark; 4 Julius Center for Health Sciences and Primary Care, University Medical Center Utrecht, Utrecht University, 3584 EA Utrecht, The Netherlands; h.w.g.derksen-2@umcutrecht.nl (J.W.G.D.); y.t.vanderschouw@umcutrecht.nl (Y.T.v.d.S.); 5 Epidemiology and Prevention Unit, Fondazione IRCCS Istituto Nazionale dei Tumori di Milano, 20133 Milano, Italy; sara.grioni@istitutotumori.mi.it; 6 Ministry of Health of the Basque Government, Sub Directorate for Public Health and Addictions of Gipuzkoa, 20014 San Sebastian, Spain; epicss-san@euskadi.eus; 7 Epidemiology of Chronic and Communicable Diseases Group, Biodonostia Health Research Institute, 20014 San Sebastián, Spain; 8 CIBER Epidemiología y Salud Pública (CIBERESP), 28029 Madrid, Spain; jmhuerta.carm@gmail.com; 9 Department of Diet, Genes and Environment, Danish Cancer Society Research Center, University of Copenhagen, Strandboulevarden 49, 2100 Copenhagen, Denmark; jytteh@cancer.dk (J.H.); annet@cancer.dk (A.T.); 10 Department of Epidemiology, Regional Health Council, IMIB-Arrixaca, 30005 Murcia, Spain; 11 Unit of Nutrition and Cancer, Cancer Epidemiology Research Program, Catalan Institute of Oncology—IDIBELL, 08908 L’Hospitalet de Llobregat, Spain; llujan@iconcologia.net; 12 Nutrition and Cancer Group, Epidemiology, Public Health, Cancer Prevention and Palliative Care Program, Bellvitge Biomedical Research Institute—IDIBELL, L’Hospitalet de Llobregat, Av. Granvia 199-203, 08908 L’Hospitalet de Llobregat, Spain; 13 Cancer Risk Factors and Life-Style Epidemiology Unit, Institute for Cancer Research, Prevention and Clinical Network (ISPRO), 50139 Florence, Italy; d.palli@ispro.toscana.it; 14 Department of Epidemiology and Biostatistics, School of Public Health, Imperial College London, London W2 1PG, UK

**Keywords:** fatty acids, dietary intake, plasma phospholipid concentrations

## Abstract

Background: The aim of this study is to determine the correlations between dietary fatty acid (FA) intakes and plasma phospholipid (PL) FA levels in the European Prospective Investigation into Cancer and Nutrition (EPIC) cohort. Methods: The dietary intake of 60 individual FAs was estimated using centre-specific validated dietary questionnaires. Plasma PL FA concentrations of these FAs were measured in non-fasting venous plasma samples in nested case-control studies within the EPIC cohort (*n* = 4923, using only non-cases). Spearman rank correlations were calculated to determine associations between FA intakes and plasma PL FA levels. Results: Correlations between FA intakes and circulating levels were low to moderately high (−0.233 and 0.554). Moderate positive correlations were found for total long-chain *n*-3 poly-unsaturated FA (PUFA) (r = 0.354) with the highest (r = 0.406) for *n*-3 PUFA docosahexaenoic acid (DHA). Moderate positive correlations were also found for the non-endogenously synthesized trans-FA (r = 0.461 for total trans-FA C16-18; r = 0.479 for industrial trans-FA (elaidic acid)). Conclusions: Our findings indicate that dietary FA intakes might influence the plasma PL FA status to a certain extent for several specific FAs. The stronger positive correlations for health-enhancing long-chain PUFAs and the health-deteriorating trans-FA that are not endogenously produced are valuable for future cancer prevention public health interventions.

## 1. Introduction

Fatty acids (FAs), the major components of lipids, are bioactive molecules involved in different metabolic pathways. Dietary FA intakes have been associated with numerous non-communicable diseases, such as cancer, neurologic disorders, cardiovascular disease, inflammatory and auto-immune diseases [1,2]. The exact pathophysiologic mechanisms causing these relationships are still unclear. However, FAs might modulate and interfere in eating behaviors [3] and disease processes [4,5,6] through their role as lipid mediators and gene transcription modulators.

In addition, the FA composition of phospholipids, the main component of all cell membranes, may alter inflammatory processes and immune functions, and might, therefore, play a role in the development of some chronic diseases [7,8,9]. FAs can influence inflammatory processes through several mechanisms [4]. Derivatives of the *n*-6 arachidonic acid (AA) have pro-inflammatory effects as precursors of signaling molecules [9]. On the other hand, derivatives of the *n*-3 FA, eicosapentaenoic acid (EPA) and docosahexaenoic acid (DHA), tend to be more anti-inflammatory [9]. In addition, trans-fatty acids (tFAs) can also be involved in development of diseases such as type 2 diabetes, cardiovascular disease [10,11,12,13] and cancer [14,15,16,17] due to their pro-inflammatory properties and inhibition of the essential omega-3 and omega-6 polyunsaturated fatty acids (PUFAs) metabolism [18,19].

Furthermore, FA concentrations are determined by many other factors such as the genetic background, smoking habits, sex, and nutrition from an early age in the human body [20,21]. For instance, it is estimated that 29% of the variance of AA in the serum phospholipids and approximately 9% of the variance of linoleic acid (LA) can be explained by genetic variants within the FADS gene, encoding the Δ5- and Δ6-desaturase enzymes [6]. 

Numerous studies have investigated the associations between dietary changes in FA intake on the FA concentrations in various lipid fractions [20,21,22,23,24,25,26,27,28]. It was previously shown that FA effects are associated with changes in the phospholipid (PL) FA composition of cell membranes [7] and that changes in dietary intake may modify this FA composition [20,21,22,23,24,25,26,27,28]. However, most of these studies have small sample sizes and often show conflicting results. In addition, they included a limited number of individual FAs, with scarce data on FA isomers. As such, the current study aims to determine the correlation between FA dietary intakes and FA plasma concentrations (including several FA isomers), using data from one of the largest cohort studies, the European Prospective Investigation into Cancer and Nutrition (EPIC). These correlation analyses, particularly for the non-endogenously synthesized FAs such as tFAs, the essential LA and alpha-linolenic acid (ALA) and long-chain PUFAs derived from these essential FAs, reflect the validity of the FA isomer intake data in EPIC.

## 2. Materials and Methods

### 2.1. Study Design and Population

The EPIC study includes 521,324 participants (about 70% are women) of which the majority are aged 35 years or older. Participants were recruited between 1992 and 2000 from 23 centers located in 10 different European countries (Denmark, France, Germany, Greece, Italy, Netherlands, Norway, Spain, Sweden and the United Kingdom (UK)). Details of the EPIC study design have been described elsewhere [29]. At recruitment, blood samples were collected and stored in liquid nitrogen (−196 °C) at the central EPIC biobank located at the International Agency for Research on Cancer (IARC, in Lyon-France), or in local biobanks (at −150 °C in Denmark, and −70 °C in Sweden). Ethical approval was obtained from the IARC Ethical Committee (IEC) in 1995 (no code available) and the respective EPIC recruiting centers. The EPIC cohort and projects undertaken within the EPIC cohort were successfully re-evaluated by the IEC on 10 May 2017. All the participants gave informed consent prior to participation. 

### 2.2. Assessment of Dietary Intake and Other Predictor Variables

The following information was collected at baseline: health status, lifestyle, anthropometry, socio-demographic characteristics, and medical history [29]. Dietary intake throughout the year before enrolment was measured by country- or center-specific validated food frequency questionnaires (FFQs) or diet histories as described previously [29,30]. The average values of used cooking fats were estimated according to each country’s habitual preparation methods as the quantification of the “hidden fats” in the diet is difficult. 

To estimate the intakes of individual FAs, the EPIC dietary intakes were matched using the National Nutrient Database for Standard Reference of the United States (NNDSR [23,25]; developed at the USDA). Intakes of FAs (isomers) were analyzed and presented as grams per day.

### 2.3. Nested Case-Control Studies with Plasma Phospholipid Fatty Acids

We used data from non-case subjects from different nested case-control studies on cancer in EPIC for whom plasma PL FAs had been assayed [31,32,33]. The total population of these combined nested case-control studies included 4923 non-case subjects (93% women). Pre-diagnostic plasma samples from breast, ovarian, pancreas and colorectal cancer cases and controls who were matched from seven countries (excluding Denmark, Norway, and Sweden) were included in the circulating PUFAs analysis. Non-cases were selected by incidence density sampling from all cohort members who were alive and free from cancer at the time of diagnosis of the matched case. Specifications for matching between cases and controls have been described elsewhere [31,32,33].

### 2.4. Measurements of Plasma Phospholipid Fatty Acids

Gas chromatography at IARC was used to determine plasma PL FA concentrations following a standardized methodology previously described and briefly summarized here [32,33]. Total lipids were extracted from plasma samples by chloroform-methanol 2:1 (*v*/*v*) containing butylated hydroxytoluene (BHT) as an antioxidant, and L-α-phosphatidylcholine-dimyristoyl-d54 as internal standard. Phospholipids were purified by adsorption chromatography on silica tubes. FA methyl esters (FAMEs) were formed by transmethylation with Methyl-Prep II (Alltech, Deerfield, IL, USA). Analyses were carried out on a gas chromatograph 7890A (Agilent Technologies, Santa Clara, CA, USA). The individual FAs were separated and identified by comparing their respective retention characteristics with those of authentic chemical standards (Sigma, St. Louis, MO, USA). Total saturated fatty acids (SFA), monounsaturated fatty acids (MUFA), PUFA, ruminant-sourced tFA (rtFA) and industrial fFA (itFA) were calculated as the sum of the given individual FAs of their family and expressed as percentages of total FAs (circulating blood FAs).

### 2.5. Individual Fatty Acids Analyzed

For both dietary intake estimation and plasma PL FAs, the following individual FAs were considered: (i) SFAs: myristic acid (14:0), pentadecanoic acid (15:0), palmitic acid (16:0), heptadecanoic acid (17:0), stearic acid (18:0), arachidic acid (20:0), behenic acid (22:0), lignoceric acid (24:0); (ii) MUFA: myristoleic acid (14:1*n*-5), pentadecenoic acid (15:1), palmitoleic acid (16:1*n*-7), heptadecenoic acid (17:1), oleic acid (18:1*n*-9), gadoleic acid (20:1*n*-11), erucic acid (22:1*n*-9), nervonic acid (24:1*n*-9); (iii) itFA: elaidic acid (18:1*n*-9/12), trans-18:2, trans,trans-18:2 and (iv) rtFA: palmitelaidic acid (16:1*n*-7/9), trans-vaccenic acid (18:1*n*-7), and conjugated linoleic acid (CLA), (v) *n*-6 PUFA: LA (18:2*n*-6), gamma-linolenic acid (18:3*n*-6), eicosadienoic acid (20:2*n*-6), di-homo-gamma-linolenic acid (20:3*n*-6), arachidonic acid (20:4*n*-6), docosatetraenoic acid, DTA (22:4*n*-6), *n*-3 PUFA; (vi) *n*-3 PUFA: ALA (18:3*n*-3ccc), EPA (20:5*n*-3), eicosatrienoic acid, ETE (20:3*n*-3), docosapentaenoic acid, DPA (22:5*n*-3), and DHA (22:6*n*-3) (see Appendix A; Table A1).

Different individual FA isomers were grouped to investigate correlations of clusters of isomers with similar characteristics (e.g., itFA and omega 3 and 6 FAs). The combination of FA isomers used in the different FA groupings are described in more depth in Appendix A.

### 2.6. Statistical Analysis

Characteristics of the study population at baseline (non-cases from nested case-control studies with PL FA data) were described (*n* = 4923) by calculating the means ± standard deviations (SDs) for continuous variables and reporting frequencies for categorical variables. Dietary intakes were expressed as grams per day (g/day) and plasma PL FAs were expressed as a percentage of total FAs (%FA) because the association between a FA, expressed as an absolute amount in the diet and absolute concentration of FAs in the blood is more variable and weaker than when circulating FAs are expressed as relative amounts [22]. Since data for dietary intake and blood values were skewed non-parametric tests were performed. Sensitivity analysis was performed with log-transformed variables but did not alter the results as such the non-transformed variables were used for ease of interpretation.

Median and interquartile ranges for dietary and plasma PL FAs were calculated. We computed Spearman rank correlations between intakes of individual and grouped SFAs, MUFAs and PUFAs and their corresponding plasma levels. Furthermore, partial Spearman rank correlations adjusted for sex, BMI (continuous), physical activity (active, moderately active, moderately inactive, inactive), smoking status (current, former, never), total energy intake (continuous), specific study (breast, ovary, colorectum, pancreas) and analytical batch were also calculated. Additionally, we also adjusted for the Epic center in a separate model (together with those variables adjusted for above). A *p*-value of 0.01 was considered to be statistically significant.

Statistical analyses were performed with Stata 14 (StataCorp LLC., College Station, TX, USA), and R version 3.6.0. (RRID:SCR_001905, Foundation for Statistical Computing, Vienna, Austria). A correlation coefficient between 0 and 0.3 (0 and −0.3) is considered to be a weak positive (negative) linear relationship; values between 0.3 and 0.7 (0.3 and −0.7) a moderate positive (negative) linear relationship and values between 0.7 and 1.0 (−0.7 and −1.0) a strong positive (negative) linear relationship.

## 3. Results

Table 1 presents certain characteristics of the study participants, which included mainly women (93%) and 4923 non-cancer cases. At recruitment, the mean age of participants was 53.90 (standard deviation (SD) = 8.32) years, while their mean BMI was 25.6 (SD = 4.31) and their mean energy intake per day was 2040 kcal/day (SD = 557). Most participants were moderately inactive (35.3%) and 56.7% never smoked.

Table 2 presents the median and inter-quartile ranges (IQR) for dietary (g/day) and plasma PL FAs (as % of total FAs). The most abundant FAs in the diet were oleic acid 25.4 g/day (IQR = 19–33.9), palmitic acid 1.15 (IQR = 0.83–1.53), LA 11.4 (IQR = 8.72–15.1), and stearic acid 5.94 (IQR = 4.43–7.78). In the circulation, the most abundant PL FA were palmitic acid 25.6 (IQR = 24.4–26.8), LA 22.2 (IQR = 20.1–24.2), and stearic acid 14.1 (IQR = 13.2–14.9).

**Figure 1:**
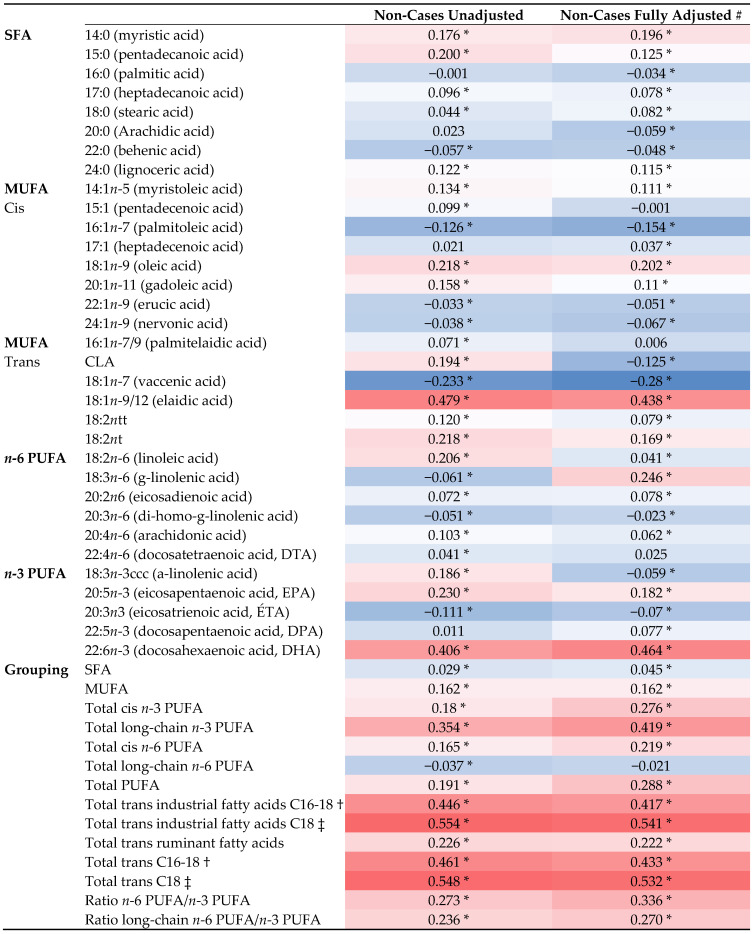
Heatmap presenting unadjusted and adjusted correlations# between dietary estimates and plasma phospholipid fatty acids for non-cases (*n* = 4923) (Darker red colours indicate stronger positive correlations and lighter red colours indicate weaker positive correlations; Darker Blue indicates stronger negative correlations and lighter blue colours indicate weaker negative correlations). Abbreviations: SFA, saturated fatty acids MUFA, monounsaturated fatty acids; PL FAs, phospholipid fatty acids; PUFA, polyunsaturated fatty acids. # Adjusted: for sex, BMI (continuous), physical activity (active, moderately active, moderately inactive, inactive), smoking status (current, former, never), energy intake (continuous), study (breast, ovary, colorectum, pancreas), and analysis batch. * Indicates *p*-value < 0.05. † Includes the sum of the following isomers Palmitelaidic acid (16:1t), Elaidic acid (18:1t), octadecadienoic trans (18:2tt) and trans 18:2t. ‡ Includes the sum of the following isomers Elaidic acid (18:1t), octadecadienoic trans (18:2tt) and trans 18:2t.

Figure 1 shows the Spearman rank (unadjusted) and partial rank correlation (adjusted) coefficients between intakes of individual FAs and their serum plasma concentrations. Significant Spearman correlations for individual FAs and their respective FA plasma concentrations were observed for LA (r = 0.206), AA (r = 0.103), DHA (r = 0.406), ALA (r = 0.186), EPA (r = 0.230), and DPA (r = 0.011).

Comparing the Spearman correlations between the grouped FA intakes and their respective FA plasma concentrations, statistically significant positive correlations were found for total SFAs (r = 0.029), total MUFAs (r = 0.162), total PUFAs (r = 0.191), trans FAs (r = 0.226), *n*-3 PUFA/*n*-6 PUFA ratio (r = 0.273) and long chain *n*-3 PUFA/*n*-6 PUFA ratio (0.236). The strongest correlations were found for tiFAs (0.446) and total tFAs (r = 0.461). Overall, statistically significant positive correlations ranged from weak (r = 0.011) to moderate (r = 0.461).

After adjusting for potential confounders, partial correlations for individual FAs and their respective FA plasma concentrations attenuated most of the unadjusted Spearman correlations coefficients: LA (r = 0.041), AA (r = 0.062), ALA (r = −0.059) and EPA (r = 0.182). The positive correlations observed for DHA (r = 0.464) and DPA (r = 0.077) remained similar.

We considered the following individual FAs for both dietary intake estimation and plasma PL FAs: (i) SFAs: myristic acid (14:0), pentadecanoic acid (15:0), pal-mitic acid (16:0), heptadecanoic acid (17:0), stearic acid (18:0), arachidic acid (20:0), behenic acid (22:0), lignoceric acid (24:0); (ii) MUFA: myristoleic acid (14:1*n*-5), penta-decenoic acid (15:1), palmitoleic acid (16:1*n*-7), heptadecenoic acid (17:1), oleic acid (18:1*n*-9), gadoleic acid (20:1*n*-11), erucic acid (22:1*n*-9), nervonic acid (24:1*n*-9); (iii) itFA: elaidic acid (18:1*n*-9/12), trans-18:2, trans,trans-18:2 and (iv) rtFA: palmitelaidic acid (16:1*n*-7/9), trans-vaccenic acid (18:1*n*-7), and conjugated linoleic acid (CLA), (v) *n*-6 PUFA: LA (18:2*n*-6), gamma-linolenic acid (18:3*n*-6), eicosadienoic acid (20:2*n*-6), di-homo-gamma-linolenic acid (20:3*n*-6), arachidonic acid (20:4*n*-6), docosatetraenoic acid, DTA (22:4*n*-6), *n*-3 PUFA; (vi) *n*-3 PUFA: ALA (18:3*n*-3ccc), EPA (20:5*n*-3), eico-satrienoic acid, ETE (20:3*n*-3), docosapentaenoic acid, DPA (22:5*n*-3), and DHA (22:6*n*-3).

Partial correlations for the grouped FA intakes and their respective FA plasma concentrations showed similar results (compared to unadjusted Spearman correlations) for SFA (r = 0.045), total MUFAs (r = 0.162), tiFAs (r = 0.417), trFAs (r = 0.222), total tFAs (r = 0.433) and long chain *n*-3 PUFA/*n*-6 PUFA ratio (r = 0.277). Stronger correlations were obtained for total PUFA (r = 0.288) and *n*-3 PUFA/*n*-6 PUFA ratio (r = 0.336). After additionally adjusting for the EPIC center, correlations attenuated (compared to unadjusted Spearman correlations) (see Appendix A; Table A2).

## 4. Discussion

Results of our study indicated low to moderately high correlations between FA intakes and circulating FA levels, ranging between (−0.233 and 0.554). Low correlations were found for total SFAs, total MUFAs and total PUFAs, while moderate positive correlations were found for long-chain *n*-3 PUFAs, with the highest correlation observed for DHA. Moderately high correlations were found for tiFAs and total tFAs. Thus, plasma FA concentrations may be related to dietary intakes, particularly for those that are not endogenously produced, including industrial trans fats and essential FAs such as LA and alpha-linolenic acid (ALA) and long-chain PUFAs like EPA and DHA which results from these essential FAs.

The results obtained in these correlation analyses are biologically plausible, considering the digestion and the metabolism of diverse FAs. Dietary FAs are metabolized in the human body (gut microbiota) after ingestion. FAs, although a minor fraction of lipids, can be oxidized and used as an energy source. Alternatively, they can be elongated and desaturated to form bioactive molecules such as signaling molecules [7]. In addition, FAs can be included in cell membranes of diverse tissues or stored in adipose tissue and thus, be compartmentalized in various lipid pools [22]. Certain FAs, such as the MUFAs of the n-9 family and the SFAs with an even number of carbon atoms can be synthesized de novo [2]. It is, therefore, not surprising that the balance between dietary intake and plasma concentrations of these FAs is weak. The weak correlations between SFA intake and their concentrations in the plasma were expected as SFAs are generally converted into MUFAs through the actions of desaturases. Low correlations have also been found in other studies; however, the correlation between total MUFA intake and serum status differed broadly across these studies (rs = 0.05–0.49) [23,24,27,34].

Essential FAs of the *n*-3 and *n*-6 families, LA and ALA, respectively, cannot be synthesized in vivo, and are, therefore, essentially derived from dietary intake. These FAs can further be processed to their long-chain derivatives, EPA/DHA and AA. However, since the conversion of the *n*-3 PUFA is not always adequate, EPA and DHA are assumed to be provisionally essential [35]. This was indicated by the moderate correlation (rs = 0.406) that was found between DHA intake and DHA plasma concentration in our study. Similar unadjusted correlations of >0.40 were found in the studies of Juan et al., Madsen et al., Hodge et al. and Ma et al. [23,27,36]. A weak correlation was found for ALA in our study. Similar low correlations for ALA were found in the study of Madsen et al. and Juan et al., [36,37]. ALA is the most rapidly oxidized and metabolized unsaturated FA and could explain why we have obtained low ALA correlations [37]. The correlation between dietary intake and plasma concentrations for LA and AA was weak and, respectively, lower and higher than evidence previously found in the literature [23,24,36,37,38]. It is noteworthy that comparisons with other studies investigating this topic are difficult due to limited evidence and differences in study design and methods used (e.g., Allison M. Hodge et al. were able to adjust their correlation coefficients for within-person variability as repeated blood samples had been collected). In addition, studies from Vyncke et al. and von Houwelingen et al. have previously shown that FAs from dietary intake and plasma concentrations are interrelated which could explain the weaker correlations observed for ALA and LA in our study [39,40].

As shown by von Houwelingen et al., dietary intake only explains 20% of the variance in the serum values of EPA and DHA [40]. This suggests that dietary intake is only one of the determining factors affecting nutritional status. As reported in genome-wide association studies, the variance in FA concentrations in blood could also be attributed to genetic variants in genes such as for instance the FADS gene cluster [5,41].

Adjusting the analyses for sex, BMI, physical activity levels, smoking habits, total energy intake, specific study and analytical batch had little impact on the correlations for individuals as well as grouped FA intakes and their respective FA plasma concentrations. However, adjusting for the EPIC center attenuated the correlations (Appendix A; Table A2). These reduced correlations observed when adjusting for the EPIC center could be due to potential lower statistical power and possibly over-adjustment [42]. Additionally, differences in FFQs used in different countries to capture dietary intake which could lead to the underestimation of trans fatty acid intakes, could also contribute to the attenuated results. Furthermore, different dietary intakes between countries and different dietary regulations, especially for tFAs, could lead to different blood values, and therefore, contribute to the observed attenuation in our results.

### Strengths and Limitations

A key strength was that data were collected from a large European sample of adults and that standardized ways, following strict procedures, were used in all blood analyses from the same sub-study. Also, several other anthropometric measurements and lifestyle assessments were collected via standardized procedures to control for a large battery of possible confounding factors. In addition, the dietary questionnaires used in all EPIC centers were validated [30]. Furthermore, several quality controls were performed to investigate the quality of the FA intake estimates isomers obtained through matching with the USDA data, which confirmed the quality of the FA data used in this study.

Limitations of this study included a high risk for various residual biases and measurement errors for both the dietary intake as well as circulating FA levels. For example, accurate collection of dietary intake data relies on the participants’ capability to recall foods and beverages previously consumed, and the translation from foods to nutrients, which is usually susceptible to errors and is less accurate. Furthermore, the quantification of the “hidden fats” in the diet was difficult to establish [43,44], especially in subjects not involved in the cooking as they are often not familiar with the food preparation methods applied and thus used fats. In addition, only one plasma sample was available per individual which does not allow adjustment for intra-individual variability in FA concentrations and as such does not necessarily fully reflect usual dietary intakes assessed via dietary questionnaires. However, when FAs were measured in the phospholipid fraction, rather than whole plasma, triglycerides (which mainly represent the FA composition of the last meal) were excluded to avoid influence from recent meals and reflect better usual FA concentrations.

Even though the same food composition table (the USDA database) was used to compare nutrient intakes between European countries, certain foods, for example, fish, consumed from different geographical origins and with different regulations might have distinct FA composition [45], which unfortunately could not be considered in these analyses. This may potentially partially explain the attenuated results after adjusting for the different EPIC centers. In addition, the different number of foods in the questionnaires of the different countries may further contribute to the attenuation of results when adjusting for the center (e.g., FFQs with fewer foods listed and fewer details may not accurately capture trans-fatty acid intakes). Potential residual and unmeasured confounding factors could not be excluded even if we adjusted for multiple covariates. Moreover, due to highly correlated dietary FAs, difficulties arise when trying to untangle their independent associations of intakes with biomarker levels.

Despite these limitations, our study suggests good quality of the dietary FA intakes computed based upon the dietary questionnaires in EPIC, particularly for the industrial trans fats and essential FAs such as DHA that are not endogenously produced.

This study is the first one to investigate correlations between dietary intakes of individual tFA from ruminant and industrial sources and their respective concentrations in plasma, showing the highest correlations with the itFAs which cannot be endogenously generated and considered as one of the health-deteriorating effects of industrial developments in food processing [18,19]. These findings together with the long-chain PUFAs (ALA and LA, respectively, which cannot be produced in vivo) suggest good validity of the dietary FA intake assessments based on the dietary questionnaire information collected in EPIC.

These analyses allow further development of guidelines regarding the optimal use of the dietary FA intake data and the PL FA concentrations in future epidemiological analyses within EPIC.

## 5. Conclusions

The present study was the first to investigate correlations between dietary FA intakes and plasma PL FA concentrations in a large-scale European cohort, allowing adjustments for several confounding factors and examinations of potential effect modifiers. Our findings indicate that dietary FA intake may influence the plasma PL FA status for several FA isomers, especially the isomers that are not or less effectively endogenously produced. These findings are important for public health strategies focusing on cancer prevention given that diet is a modifiable risk factor for cancer. Although the level of detail in dietary questionnaires is rather limited and its validity is often questioned, our results show their potential to determine the intake of FA isomers in large-scale populations where biological measurements are not possible for the whole cohort.

## Figures and Tables

**Table 1:** Selected characteristics of study participants (*n* = 4923).

	Non-Cases (*n* = 4923)
Women, *n* (%)	4614 (93.7)
Age at recruitment, years, mean (SD)	53.90 ± 8.32
BMI, kg/m^2^, mean (SD)	25.60 ± 4.31
Smoking status, *n* (%)	
never	2793 (56.7)
current	1190 (24.2)
former	845 (17.2)
missing	95 (1.9)
Physical activity, *n* (%)	
inactive	1313 (26.7)
moderately inactive	1739 (35.3)
moderately active	1051 (21.4)
active	743 (15.1)
missing	77 (1.5)
Energy intake, kcal/day, mean (SD)	2040 ± 557

**Table 2:** Median and inter-quartile ranges for dietary and plasma phospholipid fatty acids.

	Dietary Intake, g/day	PLFA, as % of Total Fatty Acids
	Non-Cases (*n* = 4923)	Non-Cases (*n* = 4923)
**SFA**		
14:0 (myristic acid)	2.68 (1.81–3.73)	0.26 (0.21–0.32)
15:0 (pentadecanoic acid)	0.05 (0.02–0.09)	0.18 (0.15–0.21)
16:0 (palmitic acid)	14.00 (10.80–17.80)	25.60 (24.40–26.80)
17:0 (heptadecanoic acid)	0.10 (0.06–0.14)	0.39 (0.35–0.44)
18:0 (stearic acid)	5.94 (4.43–7.78)	14.10 (13.20–14.90)
20:0 (Arachidic acid)	0.12 (0.08–0.18)	0.00 (0.00–0.05)
22:0 (behenic acid)	0.06 (0.04–0.11)	0.02 (0.01–0.03)
24:0 (lignoceric acid)	0.01 (0.01–0.03)	0.00 (0.00–0.00)
**MUFA**		
Cis		
14:1*n*-5 (myristoleic acid)	0.04 (0.02–0.07)	0.01 (0.01–0.02)
15:1 (pentadecenoic acid)	0.00 (0.00–0.01)	0.05 (0.03–0.07)
16:1*n*-7 (palmitoleic acid)	1.15 (0.83–1.53)	0.60 (0.48–0.75)
17:1 (heptadecenoic acid)	0.05 (0.03–0.07)	0.06 (0.05–0.07)
18:1*n*-9 (oleic acid)	25.40 (19.00–33.90)	10.10 (9.00–11.50)
20:1*n*-11 (gadoleic acid)	0.22 (0.16–0.29)	0.16 (0.14–0.20)
22:1*n*-9 (erucic acid)	0.04 (0.01–0.10)	0.03 (0.02–0.05)
24:1*n*-9 (nervonic acid)	0.00 (0.00–0.00)	0.01 (0.01–0.02)
*Trans*		
16:1*n*-7/9 (palmitelaidic acid)	0.01 (0.00–0.01)	0.23 (0.16–0.40)
CLA	0.03 (0.02–0.05)	0.20 (0.12–0.28)
18:1*n*-7 (vaccenic acid)	0.00 (0.00–0.00)	0.22 (0.13–0.33)
18:1*n*-9/12 (elaidic acid)	0.86 (0.42–1.67)	0.26 (0.18–0.42)
18:2ntt	0.00 (0.00–0.00)	0.08 (0.05–0.12)
18:2nt	0.03 (0.02–0.05)	0.04 (0.03–0.07)
***n*-6 PUFA**		
18:2*n*-6 (linoleic acid)	11.40 (8.72–15.10)	22.20 (20.10–24.20)
18:3*n*-6 (g-linolenic acid)	0.01 (0.01–0.01)	0.09 (0.07–0.13)
20:2*n*-6 (eicosadienoic acid)	0.02 (0.01–0.03)	0.36 (0.32–0.41)
20:3*n*-6 (di-homo-g-linolenic acid)	0.01 (0.00–0.01)	3.34 (2.81–3.90)
20:4*n*-6 (arachidonic acid)	0.11 (0.08–0.15)	11.00 (9.58–12.30)
22:4*n*-6 (docosatetraenoic acid, DTA)	0.01 (0.00–0.01)	0.34 (0.28–0.40)
***n*-3 PUFA**		
18:3*n*-3ccc (a-linolenic acid)	0.36 (0.23–0.57)	0.18 (0.14–0.24)
20:5*n*-3 (eicosapentaenoic acid, EPA)	0.05 (0.02–0.09)	0.97 (0.7–1.39)
20:3*n*3 (eicosatrienoic acid, ÉTA)	0.00 (0.00–0.00)	0.07 (0.05–0.08)
22:5*n*-3 (docosapentaenoic acid, DPA)	0.02 (0.01–0.03)	0.99 (0.81–1.17)
22:6*n*-3 (docosahexaenoic acid, DHA)	0.09 (0.04–0.16)	4.60 (3.75–5.64)
**Grouping**		
SFA	23.10 (17.50–29.70)	40.50 (39.60–41.40)
MUFA	26.90 (20.30–35.90)	12.80 (11.50–14.30)
Total cis *n*-3 PUFA	0.59 (0.41–0.85)	6.83 (5.73–8.29)
Total long-chain *n*-3 PUFA	0.16 (0.08–0.28)	6.63 (5.53–8.10)
Total cis *n*-6 PUFA	15.60 (11.70–21.30)	37.80 (35.90–39.70)
Total long-chain *n*-6 PUFA	0.02 (0.02–0.04)	15.30 (13.70–17.00)
Total PUFA	16.20 (12.20–22.10)	45.00 (43.30–46.60)
Total trans industrial fatty acids	0.92 (0.46–1.75)	0.74 (0.54–1.07)
Total trans ruminant fatty acids	0.04 (0.02–0.06)	0.41 (0.30–0.56)
Total trans	1.00 (0.50–1.91)	1.17 (0.88–1.64)
Ratio *n*-6 PUFA/*n*-3 PUFA	19.40 (14.40–26.90)	5.55 (4.41–6.76)
Ratio long-chain *n*-6 PUFA/*n*-3 PUFA	0.17 (0.08–0.33)	2.33 (1.81–2.89)

Abbreviations: MUFA, monounsaturated fatty acids; PLFA, phospholipid fatty acids; PUFA, polyunsaturated fatty acids; SFA, saturated fatty acids. For both dietary intake estimation and plasma PL FAs, we included the following individual FAs: (i) SFAs: myristic acid (14:0), pentadecanoic acid (15:0), palmitic acid (16:0), heptadecanoic acid (17:0), stearic acid (18:0), arachidic acid (20:0), behenic acid (22:0), lignoceric acid (24:0); (ii) MUFA: myristoleic acid (14:1*n*-5), pentadecenoic acid (15:1), palmitoleic acid (16:1*n*-7), heptadecenoic acid (17:1), oleic acid (18:1*n*-9), gadoleic acid (20:1*n*-11), erucic acid (22:1*n*-9), nervonic acid (24:1*n*-9); (iii) itFA: elaidic acid (18:1*n*-9/12), trans-18:2, trans,trans-18:2 and (iv) rtFA: palmitelaidic acid (16:1*n*-7/9), trans-vaccenic acid (18:1*n*-7), and conjugated linoleic acid (CLA), (v) *n*-6 PUFA: LA (18:2*n*-6), gamma-linolenic acid (18:3*n*-6), eicosadienoic acid (20:2*n*-6), di-homo-gamma-linolenic acid (20:3*n*-6), arachidonic acid (20:4*n*-6), docosatetraenoic acid, DTA (22:4*n*-6), *n*-3 PUFA; (vi) *n*-3 PUFA: ALA (18:3*n*-3ccc), EPA (20:5*n*-3), eicosatrienoic acid, ETE (20:3*n*-3), docosapentaenoic acid, DPA (22:5*n*-3), and DHA (22:6*n*-3).

## Data Availability

The data presented in this study are available from the corresponding author upon request. The data are not publicly available since access to the EPIC data is subject to the approval of the EPIC Steering Committee.

## Abbreviations

AA	Arachidonic acid
ALA	alpha-linolenic acid
BHT	butylated hydroxytoluene
DHA	docosahexaenoic acid
DPA	docosapentaenoic acid
EPA	eicopentaenoic acid
EPIC	European Prospective Investigation into Cancer and Nutrition
FAs	Fatty acids
FAMEs	Fatty acid methyl esters
FFQs	frequency questionnaires
LA	Linoleic acid
MUFAs	Monounsaturated fatty acids
PUFAs	Polyunsaturated fatty acids
SFA	Saturated fatty acid
tFA	Trans fatty acid

## Appendix A

**Table A1:** Variables used to compute grouped fatty acid intakes and their respective fatty acid plasma concentrations.

Groups	For Diet or Plasma	Used Isomers and Syntaxes
SFA	diet/plasma	myristic acid (14:0) + pentadecanoic acid (15:0) + palmitic acid (16:0) + heptadecanoic acid (17:0) + stearic acid (18:0) + arachidic acid (20:0) + behenic acid (22:0) + lignoceric acid (24:0)
MUFA	diet/plasma	myristoleic acid (14:1*n*5) + pentadecenoic acid (15:1) + palmitoleic acid (16:1*n*-7) + heptadecenoic acid (17:1) + oleic acid (18:1*n*-9) + gadoleic acid (20:1*n*-11) + erucic acid (22:1*n*-9) + nervonic acid (24:1*n*-9)
Total cis *n*-3 PUFA	diet/plasma	ALA (18:3*n*-3ccc) + EPA (20:5*n*-3) + eicosatrienoic acid ETE (20:3*n*-3) + docosapentaenoic acid DPA (22:5*n*-3) + DHA (22:6*n*-3)
Total long-chain *n*-3 PUFA	diet/plasma	EPA (20:5*n*-3) + eicosatrienoic acid ETE (20:3*n*-3) + docosapentaenoic acid DPA (22:5*n*-3) + DHA (22:6*n*-3)
Total cis *n*-6 PUFA	diet	LA (18:2*n*-6) + gamma-linolenic acid (18:3*n*-6) + eicosadienoic acid (20:2*n*6) + di-homo-gamma-linolenic acid (20:3*n*-6) + arachidonic acid (20:4*n*-6) + docosatetraenoic acid DTA (22:4*n*-6)
	plasma	LA (18:2*n*-6) + gamma-linolenic acid (18:3*n*-6) + eicosadienoic acid (20:2*n*6) + di-homo-gamma-linolenic acid (20:3*n*-6) + arachidonic acid (20:4*n*-6) + docosatetraenoic acid DTA (22:4*n*-6) + docosapentaenoic acid, DPA (22:5*n*6)
Total long-chain *n*-6 PUFA	diet	eicosadienoic acid (20:2*n*-6) + arachidonic acid (20:4*n*-6) + docosatetraenoic acid DTA (22:4*n*-6)
	plasma	linolenic acid (20:3*n*-6) + eicosadienoic acid (20:2*n*-6) + docosatetraenoic acid DTA (22:4*n*-6) + docosapentaenoic acid, DPA (22:5*n*-6)
Total PUFA	diet/Plasma	Total cis *n*-3 PUFA + Total cis *n*-6 PUFA
Total trans industrial fatty acids C16-18	diet	Palmitelaidic acid (16:1t) + Elaidic acid (18:1t) + octadecadienoic trans (18:2tt) + trans 18:2t
	plasma	Palmitelaidic (16:1*n*-7/9) + Elaidic acid (18:1*n* 9t) + (Trans Pufa 3 = (18:3*n* 3ctt + 18:3*n* 3tcc)) + (Trans Pufa 6 = (18:2*n* 6tc + 18:2*n* 6tt))
Total trans industrial fatty acids C18	diet	Elaidic acid (18:1t) + octadecadienoic trans (18:2tt) + trans 18:2t
	plasma	Elaidic acid (18:1*n* 9t) + (Trans Pufa 3 = (18:3*n* 3ctt + 18:3*n* 3tcc)) + (Trans Pufa 6 = (18:2*n* 6tc + 18:2*n* 6tt))
Total trans ruminant fatty acids	diet	Palmitelaidic acid (16:1t) + Conjugated linoleic acid (18:2 CLA) + Vaccenic acid (18:1*n*-7)
	plasma	Palmitelaidic acid (16:1t) + Vaccenic acid (18:1*n*-7) + conjugated linoleic acid (10t 12c) + conjugated linoleic acid (9t 11c)
Total trans C16-18	diet/Plasma	Trans industrial C18-16 + Trans ruminant
Total trans C18	diet/Plasma	Trans industrial C18 + Trans ruminant
Ratio *n*-6 PUFA/*n*-3 PUFA	diet	PUFA 6/PUFA 3
Ratio long-chain *n*-6 PUFA/*n*-3 PUFA	Diet/Plasma	PUFA 6 long chain/PUFA 3 long chain

Abbreviations: MUFA, monounsaturated fatty acids; PUFA, polyunsaturated fatty acids; SFA, saturated fatty acids. Information regarding precise syntaxes and variables used for EPIC data is available upon request.

**Table A2:** Unadjusted and adjusted *^, #^ correlations between dietary estimates and plasma phospholipid fatty acids.

	Spearman Correlation		Partial Correlation #		Partial Correlation *	
	Non-Cases (*n* = 4923)		Non-Cases (*n* = 4923)		Non-Cases (*n* = 4923)	
	r	*p*	r	*p*	r	*p*
**SFA**						
14:0 (myristic acid)	0.176	<0.001	0.196	<0.001	0.173	<0.001
15:0 (pentadecanoic acid)	0.200	<0.001	0.125	<0.001	0.111	<0.001
16:0 (palmitic acid)	−0.001	0.922	−0.034	0.019	−0.050	0.001
17:0 (heptadecanoic acid)	0.096	<0.001	0.078	<0.001	0.087	<0.001
18:0 (stearic acid)	0.044	0.002	0.082	<0.001	0.046	0.002
20:0 (Arachidic acid)	0.023	0.112	−0.059	<0.001	0.000	0.976
22:0 (behenic acid)	−0.057	<0.001	−0.048	0.001	−0.002	0.904
24:0 (lignoceric acid)	0.122	<0.001	0.115	<0.001	0.012	0.418
**MUFA**						
Cis						
14:1*n*-5 (myristoleic acid)	0.134	<0.001	0.111	<0.001	0.051	0.001
15:1 (pentadecenoic acid)	0.099	<0.001	−0.001	0.973	0.007	0.623
16:1*n*-7 (palmitoleic acid)	−0.126	<0.001	−0.154	<0.001	−0.016	0.279
17:1 (heptadecenoic acid)	0.021	0.149	0.037	<0.012	−0.012	0.416
18:1*n*-9 (oleic acid)	0.218	<0.001	0.202	<0.001	0.088	<0.001
20:1*n*-11 (gadoleic acid)	0.158	<0.001	0.110	<0.001	0.077	<0.001
22:1*n*-9 (erucic acid)	−0.033	0.021	−0.051	0.001	0.018	0.222
24:1*n*-9 (nervonic acid)	−0.038	0.008	−0.067	<0.001	−0.031	0.036
Trans						
16:1*n*-7/9 (palmitelaidic acid)	0.071	<0.001	0.006	0.699	−0.005	0.722
CLA	0.194	<0.001	−0.125	<0.001	0.013	0.379
18:1*n*-7 (vaccenic acid)	−0.233	<0.001	−0.280	<0.001	0.019	0.19
18:1*n*-9/12 (elaidic acid)	0.479	<0.001	0.438	<0.001	0.181	<0.001
18:2*n*tt	0.120	<0.001	0.079	<0.001	−0.035	0.016
18:2*n*t	0.218	<0.001	0.169	<0.001	0.018	0.212
***n*-6 PUFA**						
18:2*n*-6 (linoleic acid)	0.206	<0.001	0.041	0.005	0.037	0.011
18:3*n*-6 (g-linolenic acid)	−0.061	<0.001	0.246	<0.001	−0.026	0.08
20:2*n*-6 (eicosadienoic acid)	0.072	<0.001	0.078	<0.001	0.044	0.003
20:3*n*-6 (di-homo-g-linolenic acid)	−0.051	0.000	−0.023	0.043	−0.006	0.701
20:4*n*-6 (arachidonic acid)	0.103	<0.001	0.062	<0.001	0.057	<0.001
22:4*n*-6 (docosatetraenoic acid, DTA)	0.041	0.004	0.025	0.086	0.009	0.558
***n*-3 PUFA**						
18:3*n*-3ccc (a-linolenic acid)	0.186	<0.001	−0.059	<0.001	−0.041	0.006
20:5*n*-3 (eicosapentaenoic acid, EPA)	0.230	<0.001	0.182	<0.001	0.190	<0.001
20:3*n*3 (eicosatrienoic acid, ÉTA)	−0.111	<0.001	−0.070	<0.001	0.006	0.698
22:5*n*-3 (docosapentaenoic acid, DPA)	0.011	0.432	0.077	<0.001	0.055	<0.001
22:6*n*-3 (docosahexaenoic acid, DHA)	0.406	<0.001	0.464	<0.001	0.413	<0.001
**Grouping**						
SFA	0.029	0.044	0.045	0.002	0.003	0.858
MUFA	0.162	<0.001	0.162	<0.001	0.090	<0.001
Total cis *n*-3 PUFA	0.180	<0.001	0.276	<0.001	0.146	<0.001
Total long-chain *n*-3 PUFA	0.354	<0.001	0.419	<0.001	0.387	<0.001
Total cis *n*-6 PUFA	0.165	<0.001	0.219	<0.001	0.265	<0.001
Total long-chain *n*-6 PUFA	−0.037	0.009	−0.021	0.159	0.080	<0.001
Total PUFA	0.191	<0.001	0.288	<0.001	0.288	<0.001
Total trans industrial fatty acids C16-18 †	0.446	<0.001	0.417	<0.001	0.130	<0.001
Total trans industrial fatty acids C18 ‡	0.554	<0.001	0.541	<0.001	0.200	<0.001
Total trans ruminant fatty acids	0.226	<0.001	0.222	<0.001	0.084	<0.001
Total trans C16-18 †	0.461	<0.001	0.433	<0.001	0.101	<0.001
Total trans C18 ‡	0.548	<0.001	0.532	<0.001	0.089	<0.001
Ratio *n*-6 PUFA/*n*-3 PUFA	0.273	<0.001	0.336	<0.001	0.250	<0.001
Ratio long-chain *n*-6 PUFA/*n*-3 PUFA	0.236	<0.001	0.270	<0.001	0.360	<0.001

Abbreviations: SFA, saturated fatty acids MUFA, monounsaturated fatty acids; PL FAs, phospholipid fatty acids; PUFA, polyunsaturated fatty acids. * Adjusted for sex, BMI (continuous), physical activity (active, moderately active, moderately inactive, inactive), smoking status (current, former, never), energy intake(continuous), study (breast, ovary, colorectum, pancreas), and analysis batch. # Adjusted for all above and EPIC center. † Includes the sum of the following isomers Palmitelaidic acid (16:1t), Elaidic acid (18:1t), octadecadienoic trans (18:2tt) and trans 18:2t. ‡ Includes the sum of the following isomers Elaidic acid (18:1t), octadecadienoic trans (18:2tt) and trans 18:2t. For both dietary intake estimation and plasma PL FAs, we considered the following individual FAs: (i) SFAs: myristic acid (14:0), pentadecanoic acid (15:0), palmitic acid (16:0), heptadecanoic acid (17:0), stearic acid (18:0), arachidic acid (20:0), behenic acid (22:0), lignoceric acid (24:0); (ii) MUFA: myristoleic acid (14:1*n*-5), pentadecenoic acid (15:1), palmitoleic acid (16:1*n*-7), heptadecenoic acid (17:1), oleic acid (18:1*n*-9), gadoleic acid (20:1*n*-11), erucic acid (22:1*n*-9), nervonic acid (24:1*n*-9); (iii) itFA: elaidic acid (18:1*n*-9/12), trans-18:2, trans,trans-18:2 and (iv) rtFA: palmitelaidic acid (16:1*n*-7/9), trans-vaccenic acid (18:1*n*-7), and conjugated linoleic acid (CLA), (v) *n*-6 PUFA: LA (18:2*n*-6), gamma-linolenic acid (18:3*n*-6), eicosadienoic acid (20:2*n*-6), di-homo-gamma-linolenic acid (20:3*n*-6), arachidonic acid (20:4*n*-6), docosatetraenoic acid, DTA (22:4*n*-6), *n*-3 PUFA; (vi) *n*-3 PUFA: ALA (18:3*n*-3ccc), EPA (20:5*n*-3), eicosatrienoic acid, ETE (20:3*n*3), docosapentaenoic acid, DPA (22:5*n*-3), and DHA (22:6*n*-3).
